# WHO model of intrapartum care for a positive childbirth experience: transforming care of women and babies for improved health and wellbeing

**DOI:** 10.1111/1471-0528.15237

**Published:** 2018-05-15

**Authors:** OT Oladapo, Ö Tunçalp, M Bonet, TA Lawrie, A Portela, S Downe, AM Gülmezoglu

**Affiliations:** ^1^ UNDP/UNFPA/UNICEF/WHO/World Bank Special Programme of Research, Development and Research Training in Human Reproduction (HRP) Department of Reproductive Health and Research World Health Organization Geneva Switzerland; ^2^ Department of Maternal, Newborn, Child and Adolescent Health World Health Organization Geneva Switzerland; ^3^ Research in Childbirth and Health (ReaCH) Group University of Central Lancashire Preston UK

Globally, there is a shift in the maternal, newborn, and child health agenda from an exclusive focus on survival to the inclusion of drivers for thriving and transformation.^1^, ^2^ This shift is in line with the third Sustainable Development Goal – ensuring healthy lives and promoting wellbeing for all at all ages – and the new Global Strategy for Women's, Children's and Adolescents’ Health (2016–2030).^3^ Through research and the development of norms and standards, the World Health Organization (WHO) is supporting this global agenda by outlining a vision for high‐quality care for all pregnant women and their newborns, throughout pregnancy, childbirth, and the postnatal period.^2^ As part of this effort, WHO released new recommendations on antenatal care for a positive pregnancy experience in 2016,^4^ and recently published new recommendations on intrapartum care, again stressing the importance of a positive experience during childbirth.^5^ These recommendations go beyond the prevention of death and morbidity, as they encompass a person‐centred philosophy that includes optimising health and wellbeing for the woman and her baby.

## Why do we need to revisit intrapartum care?

Worldwide, about 140 million women give birth every year.^6^ The majority of these women and their babies are healthy and are considered to be at low risk of developing complications during labour. At the same time, for the minority of women and babies who experience complications, serious morbidity or even death can occur. Most maternity care policies recognise that all women and their babies should receive evidence‐based, equitable, compassionate, and respectful care throughout labour and childbirth; however, the reality experienced by women and babies in a multitude of settings – rich or poor – is less than positive, and access to essential interventions is not universal.

Despite decades of research, the concept of normality during labour and childbirth is not standardised. Current labour practices have seen a rapid escalation in the application of interventions to initiate, accelerate, monitor, or terminate the physiological process of labour, all with the aim of improving birth outcomes. Recent studies suggest that the benchmark for assessing normal labour progression, which was derived from studies conducted over 60 years ago, may not be appropriate for clinical decision making for individual women.^7^, ^8^ Although unnecessary labour interventions are generally more common in middle‐ and high‐income settings,^9^ the routine use of ineffective and potentially harmful labour practices are also widespread in resource‐limited settings, with the consequent misallocation of scarce resources and a further widening of the equity gap.^10^, ^11^, ^12^ On the other hand, failure to employ effective labour interventions when needed is also a recognised contributor to health inequities and poor quality of care during childbirth.^9^

In addition, the high level of mistreatment reported by women during facility‐based childbirth, and its implications for a woman's birth experience, is of significant concern.^13^ Accounts of non‐dignified and abusive care are not region‐ or culture‐specific, as they have been reported by women in low‐, middle‐, and high‐income settings.^14^

Models of intrapartum care vary considerably across settings. Depending on the healthcare system, intrapartum care service provision can be led by midwives, family doctors, or obstetricians, for example. Shared models of care also exist, and schemes like case‐loading models explicitly share professional care decisions with the woman herself; however, maternity care models are often less clear cut, as they are configured around the available human and material resources, place of birth, and philosophies of care. Although the above examples could be implemented efficiently in countries with adequate resources and well‐functioning healthcare provider training programmes, they are often challenging to implement in resource‐poor countries. Although there is promising evidence around a midwife‐led continuity of care model, it remains unclear which model is best (if any) in terms of the effects on key birth outcomes, and how feasible it is to implement the various models in different resource settings. These unresolved issues around intrapartum care call for a rethink in the fundamental approach to service provision during labour and childbirth.

## The WHO intrapartum care model

However the service is designed and delivered, there are non‐negotiable elements of good‐quality maternity care.^2^ Therefore, any strategy to improve the quality of service delivery during labour and childbirth would require a comprehensive approach that responds to all quality of care domains. The successful implementation of such a maternity service requires a model of care that gives priority to the delivery of evidence‐based practices that are acceptable to women, and which can feasibly be implemented with local adaptation. Crucially, what matters to women during labour and childbirth needs to be understood and integrated into such model of care, in order to ensure effective service design and uptake.

The synthesis of evidence supporting the development of the 2018 WHO recommendations on intrapartum care showed that women want a ‘positive childbirth experience’ that fulfils or exceeds their prior personal and sociocultural beliefs and expectations.^5^, ^15^ This includes giving birth to a healthy baby in a clinically and psychologically safe environment, with continuous emotional support from a birth companion and technically competent clinical staff. The concept was informed by the evidence that most women want a physiological labour and birth, and to have a sense of personal achievement and control through their involvement in decision making, even when medical interventions are needed or wanted. This evidence review informed the WHO guideline panel's decision to recommend selected labour and birth practices that can help women meet their goal of a positive childbirth experience.

The principles guiding the 2018 guideline, which includes 56 evidence‐based recommendations, is presented in Box1. Individual recommendations and how they affect a woman's fulfilment of a positive childbirth experience are presented in Table 1. This approach was based on the notion that through the provision of effective practices that support, and through the avoidance of ineffective and potentially harmful practices that hinder, a woman's own capabilities during the birthing process, women can be supported to achieve their desired physical, emotional, and psychological outcomes.

Box 1 **Guiding principles for intrapartum care**

- Labour and childbirth should be individualised and woman‐centred
- No intervention should be implemented without a clear medical indication
- Only interventions that serve an immediate purpose and have been proven to be beneficial should be promoted
- A clear objective that a positive childbirth experience for the woman, the newborn, and her family should be at the forefront of labour and childbirth care at all times

**Table 1:** Individual WHO recommendations and how they impact on a positive childbirth experience

Practices recommended (facilitators)	Positive childbirth experience	Practices not recommended (hindrances)
Intermittent fetal heart auscultation with a Doppler device or Pinard stethoscope; uterotonics (oxytocin or misoprostol) and controlled cord traction for the prevention of postpartum haemorrhage; delayed neonatal cord clamping; regular postnatal maternal assessment of vaginal bleeding, uterine tonus, and vital signs; intramuscular vitamin K, skin‐to‐skin contact; breastfeeding; delayed newborn bathing; postnatal maternal and newborn care for at least 24 hours in facility	Healthy mother and baby (including prevention and treatment of risks, and avoidance of death)	Routine clinical pelvimetry and cardiotocography at labour admission; continuous cardiotocography during labour; routine vaginal cleansing with chlorhexidine during labour; sustained uterine massage after birth; routine oral or nasal suction for babies with clear amniotic fluid; routine antibiotics for uncomplicated birth
Active phase starts at 5‐cm dilatation and continues for up to 12 h and 10 h; duration of second stage up to 3 h and 2 h; for nulliparous and parous women, respectively	‘Physiological labour and birth’ (without medical interventions)	Use of cervical dilatation threshold of 1 cm/h for the assessment of normal labour progression; interventions to accelerate or terminate labour before 5‐cm dilatation; perineal shaving and enema at labour admission; active management of labour; routine amniotomy, early amniotomy and early oxytocin, antispasmodics, intravenous fluids, and oxytocin for women with epidural for preventing ‘delay’ in labour; routine or liberal episiotomy; manual fundal pressure for second stage
Respectful maternity care; effective communication; 4‐hourly vaginal examination; pain relief (e.g. relaxation, manual techniques, opioids, and epidural); oral fluids and food intake, adoption of mobility, and upright position during first stage; comfortable birth position of choice regardless of epidural use, delayed pushing in women with epidural, supportive perineal techniques to reduce perineal trauma in second stage	Desire to be in control (including preserving maternal self‐esteem, competence, and autonomy, and sense of personal achievement and involvement in decision making)	Continuous cardiotocography; active management of labour; routine episiotomy; manual fundal pressure for second stage
Companion of choice, effective communication; continuity of care	Emotional support of a labour and birth companion	
Respectful maternity care; effective communication; continuity of care	Sensitive, caring, kind, skilled, and competent staff	
Postnatal care for at least 24 hours	Clinically and psychologically safe environment	Discharge prior to 24 hours

It is unlikely that any of the recommended practices can individually achieve the overall goal of a positive childbirth experience for the woman. The use of labour practices that are not focused towards the same end point can in fact have opposing effects, with no net beneficial outcome. For instance, the potential for labour companionship to increase the likelihood of spontaneous vaginal birth (with an absolute effect of 54 more per 1000), reduce the likelihood of caesarean section (with 36 fewer per 1000), and reduce the negative rating of the birth experience (with 55 fewer per 1000),^14^ to the extent observed in the systematic review included in the guideline, could be diminished if a hospital protocol dictates that cervical dilatation progressing at less than 1 cm/hour warrants intervention to expedite labour or a caesarean section. By contrast, the implementation of the principles outlined above, which allows for a rate of labour progression slower than 1 cm/hour, and encourages mobility and oral hydration, with the support from a companion of choice, could have synergistic effects that lead to a much more positive childbirth experience.

**Figure 1:**
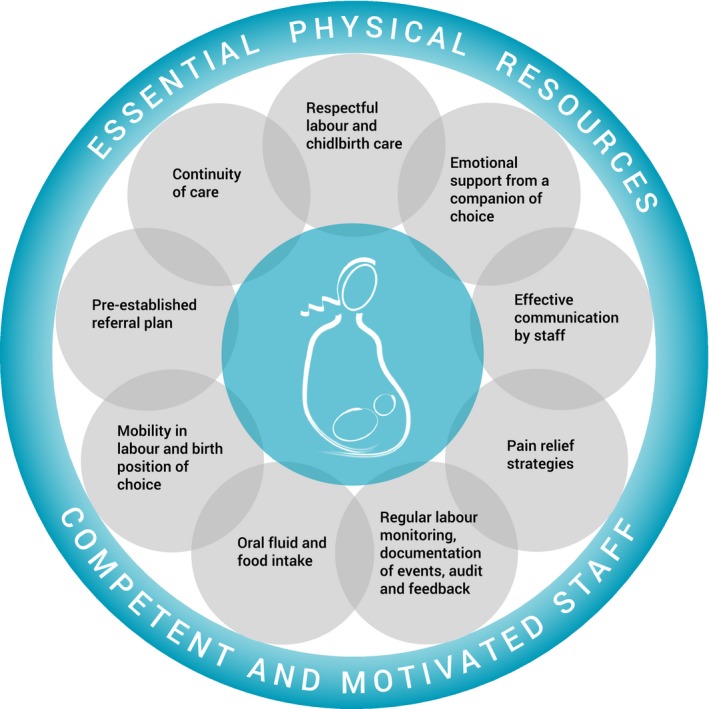
Schematic representation of the WHO intrapartum care model.

Within this context, WHO envisions intrapartum care as a platform to provide pregnant women with respectful, individualized, woman‐centred, and effective clinical and non‐clinical practices to optimize birth outcomes for the woman and her baby, by skilled healthcare providers in a well‐functioning healthcare system. To achieve this, the WHO proposes a model of intrapartum care that places the woman and her baby at the centre of care provision, and subscribes to all domains of quality of care (Figure 1). It is based on the understanding that care during labour and childbirth can only be supportive of a woman's goal when synergistic evidence‐based components are provided together. It acknowledges the differences across settings in terms of existing models of care, and is sufficiently flexible for adoption without disrupting the current organisation of care and human resources.

Healthcare systems should aim to implement this model of care in its entirety to empower all women to access the type of woman‐centred care that they want and need, and to provide a sound foundation for such care, in accordance with a human rights‐based approach. The WHO and partners are currently working on tools to support the implementation of this model at the country level, and will continue to advance research and guidance across the continuum of care to ensure that quality care within a strengthened healthcare system is a vision within the grasp of all countries.

### Disclosure of interests

None declared. Completed disclosure of interests form available to view online as supporting information.

### Contribution of authorship

The idea of this commentary was conceived by OTO. OTO, ÖT, MB, TAL, AP, SD, and AMG all contributed to the content and development of the article. OTO and ÖT wrote the first draft. All authors reviewed and agreed to the final version of this article, and approved it for publication.

### Details of ethics approval

No ethics approval required.

### Funding

None.
